# Amphibian Strategies Against Attacks by Flies: Host‐Specificity and Threats

**DOI:** 10.1002/ece3.72737

**Published:** 2026-01-13

**Authors:** Leonardo Leite Ferraz de Campos, Luiz Carlos Pinho, Selvino Neckel‐Oliveira, Ximena E. Bernal

**Affiliations:** ^1^ Department of Ecology and Zoology, Graduate Program in Ecology Universidade Federal de Santa Catarina Florianópolis Santa Catarina Brazil; ^2^ Department of Biological Sciences Purdue University West Lafayette Indiana USA; ^3^ Smithsonian Tropical Research Institute Ancón Panama

**Keywords:** ecological interactions, egg‐predators, feeding behavior, host‐use, micropredators, myiasis agents

## Abstract

Species interactions are fundamental to ecological and evolutionary processes, shaping ecosystem dynamics and driving biodiversity. Among those, interactions between flies and amphibians are common in tropical areas, yet most aspects of their ecology and evolution are understudied. Using the PRISMA method, we systematically review the literature to examine the direct and indirect threats imposed by Diptera flies attacking amphibians and the behavioral, physiological, and acoustic defenses they elicit. We delve, for instance, into the eavesdropping behavior of some dipteran species, which use anuran calls as cues for host‐seeking, and the potential impacts on frog communication systems. As flies can be disease vectors, we investigate pathogen transmission to amphibians as an indirect cost imposed by flies attacking them and examine the role of species specificity in these dynamics. Finally, we address how human activities are currently impacting these long‐established interactions between dipterans and amphibians. We focus on potential disruptions caused by habitat alteration, the presence of invasive species, and climate change. By synthesizing existing knowledge of the threats imposed by flies on amphibians, we shed light on these groups of growing conservation concern given their current escalating extinction rates. Ultimately, our findings provide valuable insights into the intricacies of species interactions and underscore the urgent need for comprehensive studies mitigating the adverse effects of anthropogenic disturbances on these clades.

## Graphical abstract

Species interactions between Diptera flies and amphibians play a significant role in tropical ecosystems, but their ecology and evolution remain understudied. This review explores the costs flies impose on amphibians, such as pathogen transmission and disruption of communication systems, and highlights the evolutionary strategies employed by both groups. It also examines how human activities like habitat alteration and climate change impact these interactions, emphasizing their conservation importance rising extinction risks.

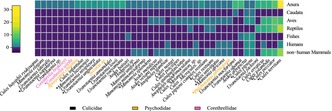

## 1 Introduction

Species interactions are the foundation of most ecological and evolutionary processes. From predator–prey relationships to mutualistic symbioses, interactions govern the flow of energy and nutrients through ecosystems (Hairston Jr. and Hairston 1993), shape the distribution and abundance of species (Wisz et al. 2013), and drive evolutionary processes (Thompson 2005). Within these complex networks, the interplay between organisms across different trophic levels stands out as a cornerstone of ecological theory and conservation practice. Predators sculpt prey populations (Schoener et al. 2001), herbivores shape plant communities (Pringle et al. 2023), and mutualistic symbioses facilitate the survival and reproduction of countless species (Stachowicz 2001). Hence, ecological interaction studies are grounded in foundational theories aiming to understand the mechanisms driving species coexistence and community dynamics (Delmas et al. 2019). Despite the importance of species interactions, however, basic knowledge about the cost imposed on each other and how anthropogenic impacts shape them remains largely inaccessible, hindering our understanding of ecological and evolutionary processes shaping interactions between species (Hortal et al. 2015).

Categorizing and measuring species interactions is challenging due to their diverse nature and context dependency (Chamberlain et al. 2014). This issue is accentuated when trying to understand the interactions of the megadiverse order of Diptera insects (true flies) with other species. Diptera represent a diverse array of species (> 167,000 described species; Bánki et al. 2023), play crucial roles in ecological processes such as decomposition (Silva et al. 2014) and pollination (Davis et al. 2023), and can engage with vertebrates in diverse ways (Waage 1979). Additionally, flies can impose strong selective pressures impacting the survival and reproduction of the species they exploit as they act as micropredators, parasites, parasitoids, and vectors of pathogens (Sarwar 2020). Despite the diverse array of species and their importance to ecosystems, most Diptera species are yet to be described (Stork 2018), and their interactions with other organisms are understudied.

Among the organisms that flies interact with, amphibians have long been crucial hosts and prey (Soghigian et al. 2023). Even though the traditional narrative presents amphibians as consumers of dipteran, their interactions are not unidirectional, and flies have evolved a remarkable arsenal of strategies to exploit amphibians. Like flies, amphibians are a diverse and cosmopolitan group with high species richness in tropical areas. Due to their reliance on terrestrial and aquatic habitats, they have evolved diverse life‐history traits (Wells 2007) and are the vertebrate group with the highest variety of reproductive modes (Nunes‐de‐Almeida et al. 2021). Amphibians thus provide multiple types of resources for flies to exploit beyond the adult stage. For instance, eggs from different species vary in composition, accompanying structures, and the habitats where they are found (Altig and McDiarmid 2007), making amphibian eggs a resource commonly used by many species of Diptera (Campos et al. 2026).

Despite their ecological importance, the interactions between amphibians and flies remain largely understudied. These interactions encompass three trophic strategies (Campos et al. 2026): (i) dipterans that prey upon amphibians' eggs and embryos (egg predators; e.g., Villa 1980), (ii) flies that cause myiasis in amphibians (myiasis agents; e.g., Silva et al. 2019), and (iii) species that rely on the blood of anurans for reproduction (micropredators; e.g., Lehane 2005). Through a systematic literature review, this study examines the direct and indirect threats imposed by flies on amphibians. By synthesizing existing knowledge on the interactions between dipterans and amphibians, we uncover the overarching patterns of flies' indirect and direct effects on amphibians' fitness, pathogen transmission, and the defense strategies that have evolved to counter such effects. From the transmission of pathogens to the evolutionary arms race between hosts and parasites, these interactions offer valuable insights into the intricacies of ecological systems. In this era of unprecedented environmental change in which both groups are experiencing high extinction rates (IUCN 2024; Wagner et al. 2021), it is critical to examine the impact of anthropogenic disturbances on species interactions. We thus evaluate current knowledge on the challenges facing interactions between amphibians and Diptera to identify patterns and venues of future research. Ultimately, this work broadens our understanding of the ecological interactions between these two clades, mainly in tropical regions where they are highly diverse.

## 2 Methods

Using the PRISMA guidelines (Moher et al. 2009; O'Dea et al. 2021; Page et al. 2021), we conducted a systematic literature review to investigate threats posed by Diptera on amphibians and the defensive mechanisms arising from these interactions. While broader syntheses on Diptera‐amphibian interactions exist (e.g., Campos et al. 2026), our review specifically focuses on threat dynamics and amphibian defensive responses. We searched Web of Science and Scopus databases using an expanded syntax that included: (1) interaction terms (eavesdropping OR “blood sucking” OR “blood feeding” OR “host use” OR “host choice” OR “host preference” OR parasit* OR eggpredator OR batracophilic OR “avoidance behavior” OR “defensive behavior” OR “defensive mechanism” OR Haematopha* OR micropredat* OR myiasis), (2) Diptera terms (insect OR dipt* OR midges OR mosquitoes OR “flies”), and (3) amphibian terms (anur* OR frog OR amphib* OR toads OR salamanders OR newt OR batrachia* OR Urodela OR caudata), restricted to titles, abstracts, and keywords. This approach was validated through iterative testing against simpler search strings (e.g., diptera AND amphibians AND interactions) and a list of previously selected foundational papers (i.e., Bernal et al. 2006; Brumpt 1934; Causey 1939; Garanin and Shaldybin 1976; Toledo et al. 2021; Villa 1980) to maximize capturing the full breadth of relevant literature without including papers where these interactions were only mentioned coincidentally.

**FIGURE 1:**
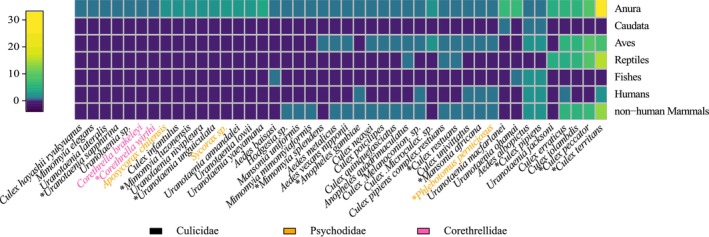
Heatmap of Diptera‐amphibian feeding interactions reported in studies using molecular tools to identify blood meals. Rows represent vertebrate groups and columns show dipteran species, with species names colored according to their family (see family legend in figure). Color intensity in each cell indicates the number of independent records for each association. Specialist vectors (e.g., *Corethrella* spp.) show exclusive association with anurans, while generalist species (e.g., *Culex pipiens* ) display broader host ranges. Species names follow original sources (see Table S1 for full references); an asterisk (*) denotes putative or confirmed vectors as listed in Table 1.

We considered only peer‐reviewed studies published before July 2023, without excluding studies based on language. In total, 846 papers were identified (Web of Science = 342; Scopus = 504). From those, 281 were excluded after deduplication, and 320 after abstract screening. The remaining 245 were full‐text read and considered when reporting an interaction between Diptera and amphibians (using observational, experimental, or molecular data) and meeting at least one of our inclusion criteria: (1) Report threats on host fitness or communication system (either directly tested or inferred by ecological, physiological or behavioral evidence); (2) Consider Diptera species as a vector of amphibians' pathogens (including either confirmed vectors or potential vectors); (3) Record flies using acoustic host‐emitted cues (either collected directly on calling males or on frog‐call traps); (4) Describe or test the defensive behavior of amphibians against flies; or (5) Report evidence of anthropogenic impacts on these interactions. We also included ten cross‐references relevant to this work, which were processed following the same inclusion criteria and restrictions used for other manuscripts. Ultimately, 74 papers written in 5 languages (English, Portuguese, French, Russian, and German) were eligible and incorporated into the qualitative synthesis. A complete list of references included in this review is provided in the (Table S1). Additionally, to further examine Diptera feeding preferences and host specificity, we extracted a subset of studies from (Campos et al. 2026) that used molecular sequencing of Diptera stomach content to determine host use based on blood meals (Table S1). By focusing on sequence‐based evidence of host use, we assessed interactions of micropredatory dipterans with anurans, revealing a broad host‐use range with variable feeding behavior strategies (Figure 1).

## 3 Results and Discussion

Diptera employ three trophic strategies when interacting with amphibians (Campos et al. 2026), resulting in varying consequences for their hosts. Micropredatory behavior (*sensu* Lafferty and Kuris 2002) is a strategy in which female flies consume a blood meal from their amphibian prey. In contrast, myiasis‐causing flies deposit eggs or larvae directly on the amphibian's skin, and the hatched larvae feed on tissues or bodily fluids. Finally, some species perform egg‐predatory behaviors, where the larvae or adult flies feed on amphibian eggs, embryos, or substances surrounding the clutches. This systematic review identified 425 records of Diptera‐amphibian interactions within the 74 papers that meet our inclusion criteria, which included 97 dipteran species from 8 families interacting with 120 amphibian species from 23 families (Table S1). Interactions were dominated by micropredators (78% of records), followed by myiasis agents (17%), and egg predators (5%).

The high number of records of trophic interactions between Diptera and amphibians identified in this review reflects the diversity of associations between species. The degree of specialization (or lack of) for most species involved in these interactions, however, is unclear. When considering micropredatory flies and focusing only on molecularly confirmed reports of host use, species fall across the entire continuum from anuran specialists to generalists that seem to be opportunistically feeding on amphibians (Figure 1). Equivalent approaches that provide a perspective of the host breadth of myiasis agents or egg predators are not available, limiting our understanding of how those strategies compare in this regard. Alas, each trophic strategy used by the flies results in distinct impacts on amphibians, which we further discuss in the following sections.

### 3.1 Direct Effects on Host‐Fitness

Dipteran‐amphibian interactions vary widely in their threats to host fitness, ranging from non‐lethal parasitism to fatal parasitoidism. Within this framework, parasitic myiasis refers to interactions where flies infest a single host with fly larvae feeding on the host's living or dead tissue, body fluids, or ingested food, causing a fitness reduction that may or may not be lethal. In contrast, parasitoidism describes interactions in which fly larvae live in their anuran hosts and are invariably lethal to their host (*sensu* Lafferty and Kuris 2002). Obligate myiasis‐causing flies, such as certain Calliphoridae and Sarcophagidae species, deposit eggs on anuran skin, with larvae subsequently migrating into host tissues and ultimately causing death (Arias‐Robledo et al. 2019; Brumpt 1934; Crump and Pounds 1985; Kelehear et al. 2020). In our dataset, Calliphorid interactions (56% of cases) disproportionately involved Bufonidae toads (57%), suggesting potential host preferences. In contrast, Chloropidae flies (e.g., *Batrachomyia* spp.) exhibit strictly parasitic myiasis, typically harboring a single larva per host and causing weight loss rather than mortality (Schell and Burgin 2001; Skuse 1889). This type of myiasis interaction was primarily documented in Hylidae and Myobatrachidae frogs (*n* = 11) and, notably, not described in Bufonidae despite this family accounting for 37% of all reported anuran myiasis cases (Table S1). Similarly, egg‐predatory and micropredatory flies reduce host fitness, with mortality likelihood increasing with infestation intensity (Camp 2006; Villa and Townsend 1983).

These contrasting outcomes of dipteran‐amphibian interactions reveal important evolutionary and ecological trade‐offs in host–parasite dynamics. Lethal parasitoidism by Calliphoridae and Sarcophagidae likely represents a specialized strategy in which fly fitness is maximized through maximum host resource depletion, despite being evolutionarily unstable due to high host mortality (Price 2020). This pattern reflects the Janzen‐Connell hypothesis which posits that specialized natural enemies disproportionately impact abundant or spatially aggregated hosts, thereby regulating host populations and promoting community diversity (Bagchi et al. 2014; Comita et al. 2014; Connel 1971; Janzen 1970). While originally developed for tropical trees, this hypothesis is strongly supported in entomological systems, including numerous studies of insect host‐parasitoid and plant‐herbivore interactions (Bagchi et al. 2014; Comita et al. 2014; Cornell and Hawkins 1995). Janzen's (1970, 1979) prediction that abundant hosts are more likely to be targeted by specialized parasites is consistent with our dataset, where toads (often ecological generalists with high local densities; Duellman and Trueb 1994) bore the highest parasitism burden. This pattern suggests that specialized dipteran enemies may similarly regulate amphibian populations, but further work examining this idea is necessary to confirm it and exclude alternative explanations (e.g., publication bias, habitat specialization).

The complete absence of Chloropidae‐Bufonidae interactions is particularly striking given Bufonidae's dominance in myiasis records. Chloropid flies that interact with frogs (e.g., *Batrachomyia* spp.) are known only for the Australasia region (Campos et al. 2026), where Bufonidae are naturally absent, which explains the lack of such association in native faunas. However, the continued absence of Chloropidae infestations on the widely introduced cane toad ( *Rhinella marina* ), deliberately released in Australia in 1935 where it became established and is highly abundant (Shine et al. 2020), suggests that factors beyond host availability, such as overcoming Bufonid chemical defenses, may also play a role (Mebs et al. 2014). These patterns suggest phylogenetic constraints (e.g., differential toxin resistance in flies or physiological responses) and ecological factors (e.g., host density, habitat use) interact in shaping species associations. Future studies evaluating comparative toxin assays, field surveys across microhabitats, and long‐term monitoring of infection fitness effects beyond mortality (e.g., reproductive costs) are necessary to better understand the intricacies of these interactions.

Egg‐predators and myiasis agents can indirectly affect the fitness of their amphibian host. The predation pressure exerted by egg‐predatory flies like *Beckeriella niger* larvae not only directly increases amphibian mortality by consuming eggs and embryos, but also affects tadpole development and survival by accelerating the time to hatch (Menin and Giaretta 2003). Premature hatching elicited by fly predation of other eggs in the same clutch likely results in underdeveloped, small tadpoles that are less likely to survive than full‐term tadpoles. This phenomenon has been shown in tadpoles that hatch prematurely due to attacks by wasps and snakes (Warkentin 1995). Additionally, the high density of predatory flies in amphibian nests could increase the mortality of eggs and embryos due to elevated ammonia resulting from the flies' excretion system (Lacey 1979). Finally, myiasis agents might also reduce the fitness of their host by attracting insectivorous birds that attempt to feed on the larvae or eggs deposited on the anuran skin (Zavadil 1997). These incidental effects warrant further investigation and may uncover rarely considered cascade effects of dipteran interactions on amphibian reproductive success.

Even though most frequently amphibians suffer a fitness reduction, some of the interactions between flies and amphibians could be mutualistic. This seems to be the case of the egg‐predatory flies from the Psychodidae family that lay their eggs proximally to or on amphibians' dead or unviable eggs and embryos. This strategy may be beneficial for amphibians under low fly larvae density conditions as it can prevent the spread of fungi and other pathogens, protecting the healthy sections of the clutch (Villa 1980). When larvae density is high, however, infections can extend to healthy eggs. Similarly, *Lucilia sericata* can opportunistically cause myiasis in amphibians (Hassine and Escoriza 2014; Whitworth et al. 2021), but its larvae usually limit themselves to feeding on dead tissue, ultimately promoting healing (Zumpt 1965). Other organisms, such as algae in egg clutches, can provide similar context‐dependent benefits to their amphibian hosts (Gilbert 1942). While these cases suggest flies could benefit amphibians under some circumstances, the fitness consequences of such associations are still unclear and deserve further investigation.

### 3.2 Trophic Transmission of Pathogens

Amphibians serve as hosts for diverse pathogens transmitted by dipterans, including protozoans, helminths, viruses, and fungi, primarily through vectors in the Ceratopogonidae, Psychodidae, Corethrellidae, and Culicidae families (Table 1). Our results identified 42 species across these four families as possible vectors of 23 pathogen taxa, with Culicidae representing 69% of all known vector species (Table 1). While Culicidae species have been disproportionately studied due to their medical importance (reviewed in Ferguson and Smith 2012), this bias has left significant gaps in our understanding of amphibian‐specific pathogen transmission. Dipteran vectors of amphibian pathogens include generalist species spanning multiple vertebrate groups (45%) and specialists only reported in anurans (55%; Table 1). As mentioned before, micropredatory flies attacking amphibians exhibit a continuum of host specificity, from strict anuran feeders (e.g., *Corethrella* spp.) to broad generalists (e.g., *Culex pipiens* ), suggesting analogous specialization in the pathogens they transmit (Figure 1, Table 1). However, most fly species are unconfirmed vectors due to limited direct evidence (e.g., molecular detection of pathogens in wild‐caught flies and anurans) and the absence of experimental infection tests.

**TABLE 1:** Dipteran species serve as potential vectors of pathogens transmitted to amphibians.

Family	Dipteran species	Microorganism taxa	Dipteran strategy	Source
Corethrellidae	*Corethrella* sp.	*Batrachochytrium dendrobatidis* (Rhizophydiales: Batrachochytriaceae)	Specialist	Toledo et al. (2021)
	*Corethrella wirthi*	*Trypanosoma* sp. (Sarcomastigophora: Trypanosomatidae)	Specialist	Johnson et al. (1993)
Culicidae	*Aedes aegypti*	*Trypanosoma rotatorium* complex (Sarcomastigophora: Trypanosomatidae)	Generalist	Ramos and Urdaneta‐Morales (1977), Bailey (1962)
	*Aedes aegypti*	*Foleyella* sp. (Nematoda: Filarioidea)	Generalist	Causey (1939)
	*Aedes aegypti*	*Foleyella dolichoptera* (Nematoda: Filarioidea)	Generalist	Gordon and Lumsden (1939)
	*Aedes triseriatus*	*Foleyella flexicauda* (Nematoda: Filarioidea)	Generalist	Benach and Grans (1975)
	*Anopheles gambiae*	*Plasmodium* (Apicomplexa: Haemosporidia: Plasmodiidae)	Generalist	Omondi et al. (2015)
	*Culex fatigans*	*Foleyella brachyoptera* (Nematoda: Filarioidea)	Generalist	Causey (1939)
	*Culex peccator*	Eastern equine encephalitis virus (Togaviridae: Alphavirus)	Generalist	Cupp et al. (2004)
	*Culex pipiens*	*Trypanosoma rotatorium* complex (Sarcomastigophora: Trypanosomatidae)	Generalist	Ramos and Urdaneta‐Morales (1977)
	*Culex pipiens*	*Foleyella* sp. (Nematoda: Filarioidea)	Generalist	Causey (1939)
	*Culex pipiens*	Sindbis virus (Martellivirales: Alphavirus)	Generalist	Omondi et al. (2015)
	*Culex territans*	*Hepatozoon clamatae* (Apicomplexa: Adeleorina)	Specialist	Kim et al. (1998), Desser et al. (1995), Ferguson et al. (2013)
	*Culex territans*	*Hepatozoon clamatae* (Apicomplexa: Haemogregarinidae)	Specialist	Kim et al. (1998)
	*Culex territans*	*Hepatozoon catesbianae* (Apicomplexa: Haemogregarinidae)	Specialist	Kim et al. (1998)
	*Culex territans*	*Trypanosoma* sp. (Sarcomastigophora: Trypanosomatidae)	Specialist	Bartlett‐Healy et al. (2009)
	*Culex territans*	*Trypanosoma rotatorium* complex (Sarcomastigophora: Trypanosomatidae)	Specialist	Desser et al. (1973)
	*Culex territans*	*Foleyella flexicauda* (Nematoda: Filarioidea)	Specialist	Benach and Grans (1975)
	*Culex territans*	*Batrachochytrium dendrobatidis* (Rhizophydiales: Batrachochytriaceae)	Specialist	Reinhold et al. (2023)
	*Culex univittatus*	West Nile virus (Amarillovirales: Kitrinoviricota: Flaviviridae)	Generalist	Omondi et al. (2015)
	*Mansonia africana*	Rift Valley fever & Bunyamwera virus (Bunyavirales: Phenuiviridae)	Specialist	Omondi et al. (2015)
	*Mansonia splendens*	Rift Valley fever & Bunyamwera virus (Bunyavirales: Phenuiviridae)	Specialist	Omondi et al. (2015)
	*Mimomyia luzonensis*	West Nile virus (Flaviviridae: Flavivirus)	Specialist	Braima et al. (2017)
	*Uranotaenia mashonaensis*	Dactylosomatidae (Apicomplexa: Adeleorina)	Specialist	Netherlands, Cook, Smit, et al. (2020)
	*Uranotaenia mashonaensis*	*Neofoleyellides boerewors* (Nematoda: Onchocercidae)	Specialist	Netherlands, Cook, Du Preez, et al. (2020)
	*Uranotaenia mashonaensis*	*Dactylosoma kermiti* (Apicomplexa: Adeleorina: Dactylosomatidae)	Specialist	Netherlands, Cook, Smit, et al. (2020)
	*Uranotaenia montana*	Dactylosomatidae (Apicomplexa: Adeleorina)	Specialist	Netherlands, Cook, Smit, et al. 2020
	*Uranotaenia montana*	*Neofoleyellides boerewors* (Nematoda: Onchocercidae)	Specialist	Netherlands, Cook, Du Preez, et al. (2020)
	*Uranotaenia montana*	*Dactylosoma kermiti* (Apicomplexa: Adeleorina: Dactylosomatidae)	Specialist	Netherlands, Cook, Smit, et al. (2020)
	*Uranotaenia sapphrina*	Eastern equine encephalitis virus (Togaviridae: Alphavirus)	Generalist	Cupp et al. (2004)
	*Uranotaenia unguiculata*	West Nile Virus (Flaviviridae: Flavivirus) & Alphamesonivirus (Mesoniviridae: Mesovirus)	Specialist	Camp et al. (2018)
Psychodidae	*Lutzomyia vexator*	*Trypanosoma* sp. (Sarcomastigophora: Trypanosomatidae)	Generalist	Anderson and Ayala (1968)
	*Phlebotomus perniciosus*	*Toscana phlebovirus* (Bunyavirales: Phenuiviridae)	Generalist	Cotteaux‐Lautard et al. (2016)
	*Sergentomyia* sp.	Dactylosomatidae (Apicomplexa: Adeleorina)	Generalist	Netherlands, Cook, Smit, et al. (2020)
	*Sergentomyia squamirostris*	*Trypanosoma bocagei* (Sarcomastigophora: Trypanosomatidae)	Generalist	Feng and Chung (1940), Feng and Chao (1943)
	*Evandromyia infraspinosa*	*Trypanosoma* sp. (Sarcomastigophora: Trypanosomatidae)	Generalist	Ferreira et al. (2008)
	*Lutzomiya gomezi*	*Trypanosoma* sp. (Sarcomastigophora: Trypanosomatidae)	Generalist	Ferreira et al. (2008)
	*Psathyromyia dendrophyla*	*Trypanosoma* sp. (Sarcomastigophora: Trypanosomatidae)	Generalist	Ferreira et al. (2008)
	*Sciopemyia servulolimae*	*Trypanosoma* sp. (Sarcomastigophora: Trypanosomatidae)	Specialist	Ferreira et al. (2008)
	*Sciopemyia sordellii*	*Trypanosoma* sp. (Sarcomastigophora: Trypanosomatidae)	Specialist	Ferreira et al. (2008)
	*Sciopemyia* sp.	*Trypanosoma* sp. (Sarcomastigophora: Trypanosomatidae)	Specialist	Ferreira et al. (2008)
Ceratopogonidae	*Forcipomyia velox*	*Icosiella neglecta* (Nematoda: Onchocercidae)	Specialist	Desportes (1941)

*Note:* Specialist strategy refers to those species that are considered amphibian specialists. In contrast, species are considered generalists when their host range includes diverse hosts beyond amphibians.

Generalist and specialist dipteran vectors likely differ in their functional roles in parasite transmission networks. Specialists (e.g., *Corethrella* spp.) may sustain enzootic cycles within amphibian populations pathogens (Table 1), whereas generalists (e.g., *Culex pipiens* , *Aedes aegypti* ) bridge transmission of generalist pathogens from amphibians to other vertebrates and vice versa (Table 1). For instance, *Phlebotomus perniciosus*, a known mammalian vector, occasionally feeds on amphibians (Figure 1), potentially facilitating cross‐taxon spillover of pathogens like *Toscana phlebovirus* (Table 1). However, vector competence needs to be assessed to better understand such potential transmission pathways. Generalists such as *Mansonia uniformis*, for instance, failed to transmit Rift Valley Fever virus after feeding on amphibians (Lutomiah et al. 2014), suggesting host‐pathogen‐vector mismatches may limit spillover risk. Limited vector competence could thus result in either dilution or amplification effects on pathogen transmission. The dilution effect occurs when increased biodiversity reduces disease risk by limiting transmission due to high encounter probability with non‐competent hosts (Keesing and Ostfeld 2021; Schmidt and Ostfeld 2001; Roberts and Heesterbeek 2018). This phenomenon may underlie cases when generalist vectors like *Culex ialombids* feed extensively on amphibians during summer months, potentially reducing arbovirus incidence through feeding on less competent amphibian hosts (Blosser et al. 2016; Wang et al. 2023). Conversely, the amplification effect occurs when generalist vectors bridge transmission between multiple competent host species, thereby increasing pathogen prevalence and distribution. While the dilution effect typically predominates in diverse ecosystems (Ostfeld and Keesing 2012), the presence of generalist vectors capable of transmitting both specialist and generalist pathogens (Table 1) adds another layer of complexity. Thus, even in species‐rich communities, highly mobile or opportunistic vectors could sustain pathogen transmission across multiple host types.

Dipteran vectors with close association with anurans, but not restricted to this group, may sustain generalist pathogens in amphibian reservoirs. *Culex territans* has been implicated in maintaining equine encephalitis viruses in anuran populations, with frogs potentially acting as overwintering reservoirs for these generalist pathogens (Tempelis and Galindo 1970; White et al. 2011). In addition, some evidence suggests that these frog‐biting mosquitoes may have evolved compatibility with specific pathogens they vector. Their physiological tolerance to *Foleyella flexicauda* results in reduced parasitemia, which seems to increase the chances of pathogen transmission by increasing the life expectancy of the mosquito (Benach and Grans 1975). Such adaptations to promote disease transmission may be the outcome of close evolutionary histories between a given vector and pathogen. The high mortality of generalist vectors infected with *Foleyella* spp., including *Aedes triseriatus* (Benach and Grans 1975), *Culex pipiens* , and *Aedes aegypti* (Causey 1939), supports the idea that broad host ranges may compromise the evolution of pathogen‐specific competence, with generalist vectors showing reduced transmission efficiency and potentially acting as dead‐end hosts for certain pathogens. Such patterns align with the resource specialization hypothesis (Futuyma and Moreno 1988), which proposes that vector‐pathogen interactions reflect trade‐offs between host range and transmission. In this way, coadaptation between pathogen, fly vector, and amphibian host could reduce antagonistic effects, potentially leading to reduced host mortality and creating tri‐trophic specialized interactions, wherein vectors specialized in amphibians interact with specialized parasites. This is illustrated by two hemiparasites, *Hepatozoon catesbianae* and *Hepatozoon clamatae*, which appear to be exclusively transmitted by *Cx. territans* to ranid frogs, hosts in which they are possibly specialized to thrive (Desser et al. 1995; Kim et al. 1998). Further work, however, is necessary to broaden our understanding of these intricate relationships and the coevolutionary dynamics (co‐specialization) between flies, the pathogens they transmit, and the amphibian hosts.

Vectors that acoustically eavesdrop on anurans calls can drive fundamentally different disease dynamics compared to non‐eavesdropping vectors counterparts, primarily due to the sex‐biased parasitism that results from their exclusive targeting of calling male frogs. Corethrellid midges, which use male frog calls for host‐seeking (Borkent 2008; McKeever 1977), create unique transmission patterns where calling males bear disproportionate parasite burdens (e.g., trypanosomes; Bernal and Pinto 2016). Despite growing evidence that female frog vocalizations are more widespread than previously anticipated (Santana et al. 2025), the lower calling output and specific acoustic structure of female anuran calls likely makes them less susceptible to eavesdropping enemies, limiting their impact on disease dynamics. In general, male‐specific targeting of eavesdropping flies attacking calling frogs mirrors phenomena observed in other acoustic sexual signal systems showing parasitism can drive evolutionary changes in signaling behavior (Zuk et al. 2006; Tinghitella et al. 2018) that can then result in rapid evolution in eavesdroppers (Wikle et al. 2025). In addition, eavesdropper‐driven disease dynamics are modulated by vector attraction to hosts signaling in mixed‐species choruses, where differential attraction to the calls of some species over others can alter transmission pathways (Trillo et al. 2023). While the associations of Dipteran vectors and the pathogens they transmit usually involve long evolutionary histories together, it has been suggested that eavesdropping flies could also vector novel amphibian disease. It has been proposed, for instance, that *Batrachochytrium dendrobatidis* (Bd) fungi are transmitted by *Corethrella* spp. (Toledo et al. 2021) and *Culex territants* (Reinhold et al. 2023), but direct experimental evidence is needed to support this conjecture. Notably, disease transmission patterns resulting from eavesdropping vectors differ from those of their non‐eavesdropping counterparts, highlighting how specialist behavior could shape unique disease dynamics between flies and frogs.

Acoustic preferences can interact with spatiotemporal variation of host and vector availability to create complex, multi‐scale transmission landscapes. The reciprocal trophic relationship between frog‐feeding dipterans and their hosts exhibits remarkable phenological synchrony, where vector emergence coincides with host breeding activity (Ferguson and Smith 2012). For instance, the summer–spring peak in *Cx. territans* attacks coincides with both anuran activity and optimal temperatures for parasite development (e.g., *Trypanosoma* spp. at 20°C–25°C; Bardsley and Harmsen 1969). This phenological match mirrors malaria‐vector systems where transmission windows align with host and vector activity (Paaijmans et al. 2010). At finer temporal scales, the infection rate of *T. rotatorium*, a common anuran parasite, varies depending on the host's activity and temperature during the day (Bardsley and Harmsen 1969). Similarly, in another system involving corethrellids biting treefrogs, the high trypanosome parasitemia in the peripheral circulatory system matches the times of frog calling activity, increasing the likelihood of transmission to other frogs at the time when corethrellids are actively seeking blood meals (Johnson et al. 1993). These multilayered spatiotemporal patterns are expected to result in complex and predictable disease landscapes, which may have important implications for understanding pathogen persistence and spillover risk in amphibian communities.

Although most flies transmit parasites to amphibians by biting them, they can also promote infection through indirect paths. The spread of pathogenic fungi to amphibian egg masses can result in additional egg, embryo, and larvae loss (Kiesecker and Blaustein 1997). Egg‐predators that promote the dispersion of fungal pathogens to these clutches, like *Megaselia randi* (Hughey et al. 2012) and *Aphiura breviceps* (Davis and Disney 2003), ultimately increase the costs of such infection. Further research targeting non‐biting routes of pathogen transmission will provide a more comprehensive understanding of these interactions and their consequences for all the parties involved. Overall, our understanding of pathogen transmission by flies to amphibians is still in its infancy and deserves further attention.

### 3.3 Effects of Eavesdroppers on Anuran Communication System

Diptera species that use anuran calls to find their host, estimated to be at least 144 species if we include all extant *Corethrella* species (Table 2), can impact the communication system of frogs. This eavesdropping behavior was first described in *Corethrella* spp. as females were observed biting calling male frogs, and their attraction to these host‐emitted cues was confirmed by trapping them broadcasting frog mating calls (McKeever and Hartberg 1980). Exploitation of frog communication systems was ultimately observed in Culicidae and Psychodidae flies too. While the evolutionary origins of this behavior remain unclear, it appears to have evolved independently among Corethrellidae and Culicidae species, possibly through convergent evolution from other frog‐biting flies' behavior (Wood and Borkent 1989). The use of acoustic signals to locate hosts might have initially evolved by co‐opting the use of hearing in a mating context (De Silva et al. 2015). We are, however, just starting to understand the driving forces and adaptations shaping this eavesdropping behavior (Pantoja‐Sánchez et al. 2023).

**TABLE 2:** Diptera species with host‐seeking behavior towards calling male anurans.

Family	Species	Source
Corethrellidae^a^	*Corethrella* spp.	McKeever and Hartberg (1980)
Culicidae	*Culex bitaeniorhynchus*	Toma et al. (2019)
	*Culex infantulus*	Toma et al. (2019)
	*Culex nigropunctatus*	Toma et al. (2019)
	*Culex territans*	Bartlett‐Healy et al. (2008); Burkett‐Cadena et al. (2008)
	*Mansonia uniformis*	De Silva et al. (2020)
	*Mimomyia luzonensis*	Toma et al. (2005); Toma et al. (2019)
	*Orthopodomyia anopheloides*	Toma et al. (2019)
	*Uranotaenia annandalei*	Legett et al. (2021); Toma et al. (2019)
	*Uranotaenia lowii*	Borkent and Belton (2006)
	*Uranotaenia macfarlanei*	Toma et al. (2005); Legett et al. (2021); Toma et al. (2019)
	*Uranotaenia mashonaensis*	Netherlands, Cook, Du Preez, et al. (2020)
	*Uranotaenia montana*	Netherlands, Cook, Du Preez, et al. (2020)
	*Uranotaenia nivipleura*	Toma et al. (2014); Legett et al. (2021); Toma et al. (2019)
	*Uranotaenia novobscura ryukyuana*	Toma et al. (2019)
	*Uranotaenia ohamai*	Toma et al. (2005); Toma et al. (2014); Legett et al. (2021); Toma et al. (2019)
	*Uranotaenia rutherfordi*	De Silva et al. (2020)
	*Uranotaenia unguiculata*	Camp et al. (2018)
	*Uranotaenia yaeyamana*	Toma et al. (2005); Toma et al. (2014)
Psychodidae	*Sycorax* spp.	Cutajar and Rowley (2020)

^a^ Except for *Corethrella mckeeveri* (Mckeever and Collness 1991), probably the entire family engages in this eavesdropping behavior.

The selective pressures imposed by eavesdropping flies on anuran communication systems are diverse and include modulating signal structure, signaling behavior, and mixed‐species signaling aggregations. Frog‐biting midges (*Corethrella* spp.), for instance, preferentially target frogs producing calls with certain acoustic properties, such as higher call rates and longer and more complex calls (Aihara et al. 2016; Bernal et al. 2006; Caldart et al. 2016; Legett et al. 2021; Meuche et al. 2016; Virgo et al. 2019). Such differences in attractiveness match the general preferences of the target receiver of those signals, female frogs (Ryan and Keddy‐Hector 1992), imposing antagonistic selective pressures on anuran calls. For example, when túngara male frogs ( *Engystomops pustulosus* ) produce complex calls by adding secondary components to their whines, they increase their signal's attractiveness to both mates (Rand and Ryan 1981) and eavesdropping frog‐biting midges (Bernal et al. 2006). This preference by the midges could be at least partly maintained due to the increased effectiveness of attack as the production of complex calls is associated with a higher density of túngara frogs (Bernal et al. 2007). While other frog‐biting flies, such as mosquitoes (Culicidae) and Sycoracinae flies (Psychodidae), likely also preferentially attack frogs producing certain call types, it is unclear whether they also impose selection on call structure, ultimately modulating signal evolution.

The complex interplay between mating signals and their unintended consequences of attracting predators and parasites reveals the multifaceted dynamics shaping signaling behavior. Eavesdropping flies, for instance, may have promoted the evolution of signal synchronization by neighboring males as signalers exploit auditory illusions to reduce attractiveness to frog‐biting midges without reducing their attractiveness to females (Legett et al. 2020, 2021). By producing their signals closely in time, one almost immediately after the other, neighboring calling males generate the precedent effect (or leader‐follower signal bias) in which frog‐biting midges perceive it as a single auditory event coming from the location of the first (leader) sound. Males calling following the calls of other males thus benefit from this acoustic illusion that results in the midges preferentially attacking the leader calling male. Furthermore, differential attacks by frog‐biting midges on males producing signals that vary in call elaboration generate cascade effects that shape male–male competition in the chorus, ultimately underscoring the pivotal role that micropredatory flies may play in sexual communication evolution (Leavell et al. 2022). In addition to the effects eavesdropping flies can generate in frog chorus dynamics, it has been proposed that defensive behaviors against frog‐biting flies could also play a role as precursors in the evolution of visual displays (Zhao et al. 2022), but further evaluation of this hypothesis is necessary (Anderson et al. 2023). The impact of eavesdropping frog‐biting midges extends to mixed‐species choruses as they alter the signaling strategies of nearby heterospecific frog signallers and may affect calling site selection (Trillo et al. 2016). Overall, eavesdropping flies can profoundly affect frog signaling behavior, shaping interactions between conspecifics and across species. We are just starting to understand how frog‐biting flies have affected the ecology and evolution of frog signals.

## 4 Defensive Mechanisms

From behavioral modifications to physiological defenses, amphibians have evolved a diverse array of tactics to mitigate the impact of dipteran attacks (Table 3). Defensive behaviors by anurans against flies have been described for the three different exploitation strategies (micropredators, egg‐predators, and myiasis agents). Amphibians use various strategies to decrease the success of myiasis‐causing fly infestation (Table 3), but these infections typically lead to the death of the host (Crump and Pounds 1985; Dodge 1968). Effective defenses thus are focused on preventing infection by consuming approaching adult flies or larvae and eggs deposited on the skin (Brumpt 1934; Zavadil 1997), performing movements such as brushing the nostril with the forelimbs or rubbing on the ground or molting and eating the skin in response to deposited eggs (Zavadil 1997). Amphibians also submerge themselves in water, attempting to suffocate the larvae or the eggs (Eaton et al. 2008), which is in line with the idea that myiasis is more common for terrestrial amphibians (Hagman et al. 2005). Chemical defenses provide a crucial deterrent, with many amphibians secreting a diverse arsenal of bioactive compounds, including some that can alter insect behavior. Skin peptides of species like *Litoria caerulea* can act as insect repellents (Williams et al. 1998), and toxins like pumiliotoxins present in poison frogs exhibit targeted toxicity against mosquitoes (Weldon et al. 2006). While most work has focused on examining the effect of anuran secretions on adult mosquitoes, skin secretions of some Amazonian frogs (*Lepadodactylus knudseni* and *Phyllomedusa vaillantii* ) are also lethal to mosquito larvae (Trindade et al. 2014). However, some specialized flies have evolved countermeasures, such as *Lucilia bufonivora*, which is tolerant to bufadienolides produced by their host toads (Mebs et al. 2014). Unlike myiasis in other terrestrial vertebrate groups, amphibians can be infested even on healthy skin, and the infection affects both non‐toxic and toxic species (Bolek and Coggins 2002; Bolek and Janovy 2004; Crump and Pounds 1985; Hagman et al. 2005). Such high vulnerability is likely behind the multifaceted nature of defenses against myiasis in frogs.

**TABLE 3:** Main defensive behaviors deployed by amphibians against dipteran attacks.

Dipteran strategy	Dipteran species	Anuran defensive behavior	Anuran species	Source
Eggpredator	—	Parental Care	—	Seshadri and Bickford (2018)^a^
Eggpredator	*Beckeriella niger*	Hatching Earlier	*Engystomops cuvieri*	Menin and Giaretta (2003)
Eggpredator	—	Subterranean Chamber	*Leptodactylus furnarius* ; *Adenomera* sp.	Menin and Giaretta (2003)^a^
Myiasis agent	*Lucilia bufonivora*	Change Skin^b^	*Bufo Bufo*	Zavadil (1997)
Myiasis agent	*Lucilia bufonivora*	Eat the eggs and larvae^c^	*Bufo Bufo*	Zavadil (1997)
Myiasis agent	*Lucilia silvarum*	Submerge in water	*Rana sylvatica*	Eaton et al. (2008)
Myiasis agent	*Lucilia bufonivora*	Mechanical movements	*Bufo Bufo*	Zavadil (1997)
Myiasis agent	*Lucilia cuprina*; *Calliphora stygiahoridae*	Peptides on Skin	*Litoria caerulea*	Williams et al. (1998)
Micropredator	*Corethrella* spp.; *Culex territans*	Mechanical movements	—	McKeever (1977); Bernal et al. (2006); Gould and Valdez (2024)
Micropredator	*Culex annulirostris*	Peptides on Skin	*Litoria caerulea*	Williams et al. (2006)
Micropredator	*Corethrella* spp.	Call synchronization	*Smilisca sila* ; *Dendropsophus ebraccatus*	Legett et al. (2021); Ruether et al. (2022)

^a^ Indicate behaviors that are proposed to be defensive strategies against flies, but have not been confirmed. The hyphen (–) represents undetermined taxa.

^b^ Change Skin refers to molting behavior, where the amphibian sheds its skin to remove fly eggs or larvae.

^c^ Eat the eggs and larvae describes the amphibian consuming the eggs or larvae of the dipteran parasite from its own body.

Amphibians use indirect and direct defense strategies against egg‐predatory dipteran flies (Table 3). In response to perceived threats from dipteran predators, premature hatching can minimize consumption during vulnerable developmental stages. Tadpoles of *Engystomops cuvieri*, for instance, emerge earlier in nests subject to predation by maggots of the fly *Beckeriella niger* (Menin and Giaretta 2003). Although premature hatching could decrease the survival of tadpoles due to their small size and less developed stage (Warkentin 2011), it is an effective short‐term defensive strategy to avoid egg predation (Warkentin and Caldwell 2009). In frogs with parental care, guarding parents can protect their eggs against Diptera predators to prevent infestation (Seshadri and Bickford 2018). In leptodactylid frogs, such as *Leptodactylus furnarius* and *Adenomera* sp., which construct subterranean chambers for their foam nests, the eggs and tadpoles are protected from dipteran flies (Menin and Giaretta 2003). Given that this nesting behavior effectively deters egg‐predatory dipteran infestation, this suggests that egg‐predatory flies may have been an important driving force for the evolution of subterranean nest chambers.

Micropredatory dipteran flies employ efficient strategies to locate and feed on their amphibian prey. In response, amphibians have evolved various defensive mechanisms (Table 3) aimed at either directly avoiding attacks through limb movements (Crans 1970; Gould and Valdez 2024), or producing skin substances to repel them (Weldon et al. 2006; Williams et al. 2006). Furthermore, given that a large proportion of frog‐biting dipterans specialize in host‐emitted mating calls (Table 2), calling frogs deploy diverse tactics to deter their attacks. Individuals from many anuran species, for instance, use limb movements to deter fly attacks (Bernal et al. 2006; Gould and Valdez 2024; McKeever 1977). As mentioned before, call synchronization among neighboring frogs within a chorus can also reduce fly attacks to males producing “following” calls (Legett et al. 2019, 2021). Given that signaling heterospecific frog neighbors can alter predation risk by *Corethrella* flies (Ruether et al. 2022; Trillo et al. 2016), calling site selection relative to conspecifics and heterospecifics in the chorus is expected to be a strategy used to call from safer spaces. Further work, however, that directly examines calling site selection relative to other signalers in the chorus with diverse predation risk from frog‐biting midges is still needed to examine this prediction. The strategies used by anurans to defend themselves from frog‐biting flies suggest they impose strong selective pressure. These include costs imposed by blood loss (Camp 2006), disease transmission (Bernal and Pinto 2016; Johnson et al. 1993; Meuche et al. 2016), and reduced female attraction (De Silva et al. 2015; Leavell et al. 2022).

As anurans deploy defenses against fly attacks, dipterans are under pressure to find alternative strategies that circumvent them. In some cases, for instance, flies only attack the frogs at the moment when they are calling, potentially avoiding consumption and defensive limb movement attacks by the frog (Netherlands, Cook, Du Preez, et al. 2020). It is also possible that biting site selection is modulated by defense strategies favoring areas more difficult to reach by the swatting frogs, but there is no evidence yet that this is the case. Overall, amphibians employ diverse defensive tactics, while dipterans are expected to adapt to exploit vulnerabilities in these defenses. Our understanding, however, of such complex co‐evolutionary dynamics between these two groups is limited.

## 5 Anthropogenic Impacts

Human‐mediated effects can disrupt ecological relationships between Diptera and amphibians, resulting in the emergence of novel interactions (Blosser et al. 2016; Richards et al. 2006) and, in some cases, the elimination of interactions (McMahon et al. 2017). Such disruption of species interactions could also change disease dynamics when disease vectors are involved. Although there is no documented information about these changes, host‐pathogen interaction dynamics can be impacted by environmental temperature shifts, changes in host susceptibility to infection, and ultimately affect both host and pathogen survival (Cattadori et al. 2005). Amphibians are the most threatened vertebrate class (IUCN 2024). Their high extinction rates during the last two decades of the previous century have been mainly driven by habitat loss and disease. Climate change has recently become a major threat to this group, as it threatens almost 29% of species, mainly in tropical areas (Luedtke et al. 2023). Given the large proportion of amphibian species with population declines, species that rely on them for survival and reproduction, like the flies described in this review, are expected to be affected due to limited host availability.

Changes in host‐use can also occur with the introduction of anurans or dipteran species to a given area. The presence of zoos, for instance, potentially serves as hubs for the transmission of diseases between wild amphibian populations, captive animals, and humans (Heym et al. 2019; Tuten et al. 2012). Synanthropic species such as the fly *Megaselia scalaris* (Phoridae) exploit opportunities in urban settings and were found biting frogs from a pet shop (Zwart et al. 2005). The introduction of invasive amphibians or dipterans can also impact ecological dynamics by altering host‐pathogen interactions. Given that invasive amphibian species have high abundance (Gersava et al. 2020), contact rates between dipteran flies and these invasive hosts could alter the interactions between native dipteran and amphibians. The native mosquito *Mimomyia elegans*, for example, interacts with invasive cane toads ( *Rhinella marina* ) in Australia (Beurden 1980). It is unclear, however, how this novel host‐vector interaction may have changed pathogen dynamics in this area. Introductions of species may trigger cascade effects resulting from novel host‐vector interactions, which could result in changes in pathogen prevalence or the emergence of new diseases. Invasive species such as cane toads and American bullfrogs ( *Lithobates catesbeianus* ) can act as reservoirs for a wide range of pathogens that affect native species (Atkinson and Savage 2023).

Non‐native dipterans capable of feeding on native amphibians can further disrupt interactions between native dipterans and amphibians. Species such as *Aedes aegypti* , known for their high invasiveness and ability to feed on various vertebrate groups, including amphibians (Figure 1), potentially spread diseases between wild and urban populations. Human‐altered landscapes may exacerbate spillover risks by increasing contact opportunities between generalist vectors, like *Aedes aegypti* , and amphibian populations. Introduced dipterans often exhibit opportunistic feeding behavior and can thus serve as bridges between wild amphibian pathogen reservoirs, livestock, and human populations. Such risk of spillover is of particular concern in areas where native host diversity has declined (Blosser et al. 2016). Furthermore, invasive species may impact specialist dipteran species that depend exclusively on amphibians for survival and reproduction by competing with them for access to hosts. Therefore, the introduction of non‐native species potentially results in significant implications, underscoring the necessity for comprehensive studies on changes in host‐use patterns and host‐pathogen interactions associated with the arrival and establishment of invasive species.

In comparison to the effects of invasive species, less is known about how other anthropogenic changes impact the interactions between flies, frogs, and associated pathogens. Artificial light at night and noise pollutants created by urbanization, for instance, can also influence the attractiveness of frog‐biting dipteran specialists to amphibian calls (McMahon et al. 2017). These sensory pollutants can alter the distribution of hosts and vectors, intensifying some interactions and extinguishing others. However, with the imminent climate instability, it is reasonable to infer that fluctuations in temperature and humidity, known to influence both frog‐biting dipteran breeding rates (Cotteaux‐Lautard et al. 2016) and anuran phenology (Sheridan et al. 2018; Wu et al. 2024), could also impact these interactions. Dry periods are often related to increased incidence of myiasis in some anuran species (Crump and Pounds 1985; Zumpt 1965), which can significantly affect host survivorship in certain years (Garanin and Shaldybin 1976). Thus, with the expected increasing frequency of dry periods due to climate change in some areas (Ritchie et al. 2022), the impact of myiasis infestation on amphibian species is expected to escalate. Climate‐driven shifts in vector distributions (e.g., *Culex pipiens* expansion; Medlock et al. 2012) may further destabilize host‐pathogen networks, especially in populations in which generalist vectors bridge amphibian and human populations. Overall, anthropogenic alterations could have major impacts on the interactions between dipterans and amphibians ultimately further amplifying the high levels of extinction currently experienced by both groups (IUCN 2024; Wagner et al. 2021).

## 6 Conclusions and Future Directions

The intricate relationships between Diptera and amphibians exemplify how these interactions could be driving evolutionary and ecological processes across scales in these taxa. While myiasis parasitoid interactions often result in severe declines in host fitness, similar to predators of eggs, flies causing myiasis can also paradoxically result in benefits to the amphibians they attack. When considering micropredators, the transmission of pathogens by dipteran vectors introduces an additional layer of complexity, with specialist and generalist vectors differentially influencing disease dynamics. Given the selective pressures imposed by flies on amphibians, diverse defensive strategies have evolved to minimize their attacks, ultimately highlighting the important role that dipterans have played in shaping the behavior and biology of amphibians through evolutionary time.

Several research gaps remain apparent. Only 28% of studies came from tropical regions despite these areas containing the highest species diversity (Myers et al. 2000). While no language restriction was incorporated in our systematic review, the predominance of research investigating species from temperate regions (particularly North America and Europe) in our dataset likely underrepresents the true diversity of these interactions. This geographic skew mirrors broader biases in ecological research funding and publication patterns (Hortal et al. 2015), leaving critical knowledge gaps for biodiverse but uninvested regions. For instance, while phenological matching between dipterans and amphibians is documented in temperate zones (e.g., *Trypanosoma* transmission peaks coinciding with anuran activity), the consequences of climate‐driven phenological shifts remain unstudied in tropical systems where rainfall and temperature seasonality tightly couple amphibian breeding with dipteran activity (Bardsley and Harmsen 1969; Johnson et al. 1993), a synchrony increasingly disrupted by climate warming. Additionally, most putative dipteran vectors of amphibian disease lack experimental transmission studies, and, to our knowledge, no research has examined how climate warming alterations in species phenology ultimately modulate pathogen‐dipteran‐amphibian interactions. Likewise, urbanization may amplify spillover risks by favoring generalist vectors (e.g., *Aedes aegypti* , *Culex pipiens* ), while habitat fragmentation could disrupt acoustic eavesdropping systems critical for frog‐biting fly host‐searching. Such changes would have cascading implications, as dipteran‐driven selection on amphibian communication traits may be disrupted by sensory pollutants like artificial light or noise pollution.

Understanding and monitoring the interactions between flies and amphibians is crucial for conservation efforts, especially given the current global decline of both groups (IUCN 2024; Wagner et al. 2021). These interactions are essential for biodiversity conservation, as amphibians serve as vital resources for many dipteran species and the pathogens they carry. Despite the importance of these interactions, they have long been overlooked, and our current understanding of those associations is limited. Recognizing the roles these species play in ecosystem dynamics is essential for safeguarding biodiversity in the face of current global changes. Bridging this gap requires integrating interaction networks into conservation planning, particularly for specialized systems. By shedding light on the complexities of these interactions, we hope to inspire further research on this topic to promote conservation efforts that can work towards preserving these intricate relationships.

## Author Contributions

**Leonardo Leite Ferraz de Campos:** conceptualization (lead), data curation (lead), formal analysis (lead), funding acquisition (equal), investigation (lead), methodology (lead), project administration (lead), resources (lead), validation (lead), visualization (lead), writing – original draft (lead), writing – review and editing (lead). **Luiz Carlos Pinho:** validation (equal), writing – review and editing (supporting). **Selvino Neckel‐Oliveira:** funding acquisition (lead), supervision (supporting), writing – review and editing (supporting). **Ximena E. Bernal:** conceptualization (equal), funding acquisition (equal), methodology (equal), project administration (lead), supervision (lead), writing – original draft (equal), writing – review and editing (equal).

## Funding

This work was supported by the National Science Foundation Division of Integrative Organismal Systems, (IOS‐1433990). Coordenação de Aperfeiçoamento de Pessoal de Nível Superior, Finance Code 001.

## Conflicts of Interest

The authors declare no conflicts of interest.

## Supporting information


**Table S1:** Species Records. This supplementary table provides a comprehensive list of Interacting species records included in “Campos et al. 2026—Diptera interactions with amphibians: A review of the ecological impacts”, incorporating.

## Data Availability

All the data generated or analyzed during this study are provided in the Supporting Information S1. The data will be deposited at the Purdue University Research Repository (PURR), an online data repository that is publicly available for download.
